# Functional recovery with peripheral nerve block versus general anesthesia for upper limb surgery: a systematic review protocol

**DOI:** 10.1186/s13643-019-1204-4

**Published:** 2019-11-11

**Authors:** Jennifer Héroux, Emilie Belley-Côté, Pablo Echavé, Marie-Josée Loignon, Pierre-Olivier Bessette, Nicolas Patenaude, Jean-Patrice Baillargeon, Frédérick D’Aragon

**Affiliations:** 1 Université de Sherbrooke, Sherbrooke, Québec Canada; 2 McMaster University, Hamilton, Ontario Canada

**Keywords:** Upper limb surgery, Peripheral anesthesia, Regional anesthesia, Nerve block, Brachial plexus, General anesthesia, Postoperative recovery, Functional recovery

## Abstract

**Background:**

Peripheral nerve block is a single injection that inhibits the transmission of peripheral nerve impulses to the central nervous system. The inhibition of the nociceptive impulse may decrease the occurrence of muscle spasm following mobilization postoperatively. This mechanism may contribute to a better functional recovery following upper limb surgery. This systematic review will investigate the impact of peripheral nerve block on functional recovery after an upper limb surgery.

**Methods:**

We will search studies comparing peripheral nerve block to general anesthesia for upper limb surgery in the following databases: CENTRAL, MEDLINE (Ovid), CINAHL, EMBASE, and Scopus. In duplicate, independent reviewers will assess eligibility, evaluate risk of bias, and abstract data on type of peripheral nerve block and functional outcome. Where possible, we will pool results using a random effects model. For each outcome, we will assess the quality of evidence using GRADE methodology.

**Discussion:**

We aim to summarize the available evidence comparing functional recovery with peripheral nerve block versus general anesthesia for upper limb surgery. These data will inform the design of a trial on the topic.

**Systematic review registration:**

PROSPERO CRD42018116298

## Background

### Description of the condition

Orthopedic surgery of the upper limb can be performed under general anesthesia or peripheral nerve block. General anesthesia is carried out with the injection of multiple anesthetic agents to have the patient unconscious and insensible to painful stimulation. General anesthesia is a suitable option for anxious patients, for long procedures, and for patients with contraindications to regional anesthesia [1]. Peripheral nerve block involves injecting local anesthetics around the nerves involved in the part of the body that will be operated on, aiming to inhibit the transmission of peripheral nerve impulses to the central nervous system for the procedure to happen without the patient feeling painful stimuli [2].

Of upper limb surgeries, 20% are performed under peripheral nerve block and 80% are performed under general anesthesia [3]. General anesthesia is safe, economical, and familiar to both the patient and the anesthesiologist. For multiples fractures, general anesthesia helps to alleviate rapidly the pain [4–6].

The advantage of peripheral nerve blocks is that they provide analgesia and immobilization to the target limb while avoiding the side effects of general anesthesia which include nausea/vomiting, sore throat, fatigue, and prolonged hospital stay. For elderly patient, it decreases the incidence of post-operative delirium. In case of severe trauma, vasoplegia from the sympathetic blockage can optimize blood flow and improve surgical outcome [7]. Furthermore, the inhibition of the nociceptive impulse, responsible for muscle spasm following mobilization postoperatively, is hypothesized to improve pain relief and recovery for the procedure following peripheral nerve block. In fact, peripheral nerve blocks are associated with improved analgesia on the day of the surgery with a longer time to first opioid consumption (regional anesthesia (RA) 97.6 ± 50.2 min; general anesthesia (GA), 29.9 ± 22.8 min; *P* < 0.001), reduced dose of opioid (RA, 7.3 ± 15.2 mg of oral morphine equivalent; GA, 22.8 ± 18.1 mg; *P* < 0.01), and shorter hospital stay (RA, 100.4 ± 45.6 min; GA,142.6 ± 49 min; *P* < 0.001) [8].

The functional recovery, following an orthopedic surgery, is an important part of the success of the surgery. The World Health Organization (WHO) defines a disability as “a multidimensional concept related to the dynamic interaction between body functions and structures, activity limitations, and participation restrictions alongside environmental and personal factors” [9]. Functional recovery is a broad outcome with no specific definition: it may vary from the ability to return to work or normal activities following the surgery to a complex scoring tool including range of motion, pain, and ability to perform daily activities [10].

In the literature, there are approximatively 144 instruments or psychometric evaluations to evaluate functional recovery for surgery of the upper limb: 47% are patient reported and 53% are clinician based. Patient-reported outcome measurement is an important part of the functional recovery. There is a disparity following upper limb surgery between the biomedical evaluation by the surgical team, which includes the range of motion following a surgery or the fracture union in case of trauma, and the biopsychosocial aspect of the recovery. Emotion, social or financial status, secondary gain, or pain have shown to have more influence on the persistence of the disabilities and functional recovery than the type of surgery or fracture [9].

Randomized controlled trials published comparing peripheral nerve block to general anesthesia on functional recovery after upper limb surgery have reported contradictory results that do not inform clinicians, patients, and health administrators about optimal practice. A systematic review evaluating the most performed peripheral nerve blocks in regional anesthesia concluded that peripheral nerve blocks provided better anesthesia as assessed with pain scores and opioid consumption. This review included less information on functional recovery following surgery with only one randomized controlled trial reporting on this outcome with the supraclavicular approach [11]. While a gap exists between the scarce available data for upper limb surgery, studies evaluating functional recovery after knee surgery favor peripheral nerve block anesthesia [12]. If regional anesthesia improved functional outcomes, it would lead to its wider adoption in clinical practice and a greater emphasis on regional anesthesia in training. On the other hand, if it does not impact functional outcomes, its use may decrease as it increases anesthesia set up time [12–14].

### Description of the intervention

Local anesthetics inhibit the action of the voltage-gated sodium channels by interacting with specific receptors in the inner vestibule of the sodium channel, thus preventing depolarization. This inhibition blocks conduction and prevents the nerve influx from propagating. In clinical practice, according to the type of fiber and the concentration of local anesthetic, blockade progresses from the autonomic nervous system to sensory anesthesia and finally skeletal muscle paralysis [2].

There are four approaches to block the brachial plexus for upper limb surgery: interscalene, supraclavicular, infraclavicular, and axillary. The interscalene block is used mainly for shoulder surgery. The supraclavicular, infraclavicular, and axillary blocks are indicated for surgery of the hand or arm. The approaches are named after the adjacent structures when performing the block [15].

### How the intervention might work

We hypothesize that (1) early postsurgical mobilization is associated, in the short and long term, with improved range of motion and functional recovery and (2) peripheral nerve blockage, compared to general anesthesia alone, favors early mobilization through better pain control [12].

In addition, peripheral nerve blocks are thought to have anti-inflammatory effects. By blocking C-fiber activation and reducing cytokine production, they may reduce tissue inflammation and the associated pain. As cytokines are responsible for the development of postoperative hyperalgesia, reducing their production may decrease postoperative hyperalgesia. By blocking sympathetic nerve activity, peripheral nerve blocks may also diminish inflammation [12].

### Why is it important to conduct this review?

In 2004, McCartney et al. conducted the first randomized trial comparing axillary brachial plexus block to general anesthesia for ambulatory hand surgery in 100 patients. The hypothesis was that peripheral nerve block, which provides good early pain control, may help provide better pain control and decrease pain-related disability up to 14 days after the surgery. Outcomes included (1) pain score using a visual analogue scale (VAS), (2) opioid consumption, (3) pain disability index and (4) patient satisfaction at post-operative days 1, 7, and 14. Peripheral nerve block significantly improved analgesia on the day of the surgery, but at days 1, 7, and 14, there was no significant difference in the pain level, opioid use, pain disability index, and satisfaction with anesthesia. However, this study is limited by the fact that their pain disability index was focused on pain and did not evaluate functional recovery [8].

Hadzic et al. conducted a 52-patient randomized controlled trial of infraclavicular nerve block versus GA for day surgeries of the hand and wrist. On the day of surgery and at 72 h, the pain score was lower in the peripheral nerve block group (day of the surgery VAS > 33% with RA versus 43% with GA; pain at 72 h for RA versus GA: low 88% vs 58%, moderate 13% vs 38%, high 0% vs 4%). No significant difference was observed at 24 and 48 h. However, the study had a short follow-up and did not evaluate the time to mobilization post-surgery nor functional recovery [7].

Kessler et al. conducted a systematic review evaluating blocks in RA of the upper limb, lower limb, and trunk; they found 28 studies evaluating the effectiveness of RA as assessed by postoperative pain, opioid administration, request for GA, and patient satisfaction. Secondary outcomes included functional recovery and complications related to peripheral regional anesthesia. For all four upper limb approaches, the primary outcome was achieved. Only 1 study of the interscalene approach evaluated functional recovery with the Constant score, a multi-modal scale integrating pain score, range of motion, daily activities, and strength, and this evaluation was only available for the duration of the hospital stay. The Constant score was significantly reduced with peripheral nerve block; however, they used a continuous catheter, providing long-term anesthesia, rather than a single-shot [11].

We will perform a systematic review and, if possible, meta-analyze the result of randomized controlled trials that have evaluated functional recovery after upper limb surgery, including the four peripheral nerve block approaches, at short, mid, and long term.

Since the publication of Kessler et al., many trials were published. In addition, we will include trials in which multiple upper limb block approaches were used or multiple local anesthetic agents were used, possibly providing more articles [11].

### Research question

In patients undergoing upper limb surgery, does peripheral nerve block improve functional recovery in the short, medium, and long term when compared to GA?

## Materials and methods

The following protocol is based on the Preferred Reporting Items for Systematic Reviews and Meta-Analyses (PRISMA) statement and is registered with PROSPERO (ref #: CRD42018116298).

### Study eligibility criteria

#### Type of studies

We will include randomized controlled trials (RCT) and controlled observational studies assessing the use of peripheral nerve block compared to GA for upper limb surgery. Retrospective or prospective observational studies (cohort or case-control) with a control group will be eligible. We will exclude case reports and case series [16]. There will be no restrictions based on language, methodological quality, year of publication, or the status of publication.

#### Types of participants

The population of interest consists of adult patients (≥ 18 years) undergoing surgery of the upper limb. We will include any type of surgery of the upper limb (i.e., arthroplasty, open reduction internal fixation (ORIF), reimplantation, mass/tumor excision). We will exclude animal studies.

#### Type of interventions

The intervention of interest is brachial plexus block (supraclavicular, infraclavicular, axillary, and interscalene) performed for a surgery. We will exclude studies in which there is a concomitant use of both GA or local infiltration because of the impossibility to evaluate the portion of the functional recovery attributable to the peripheral nerve block and GA. If a study included a proportion of patients with concomitant GA or local infiltration, we will either include only the patients who did not (if these data are available) or include the study if less than 20% of patients had concomitant GA or infiltration.

#### Types of outcome measures

We will include studies with at least one of following study outcomes:

- Functional recovery;
- Range of motion;
- Patient satisfaction regarding the anesthetic technique used;
- Quality of life following the surgery;
- Time interval from surgery to return to work.

If pooling is appropriate, we will meta-analyze the results separately for patient- and clinician-based evaluation and at 3 timepoints post-surgery: short term (< 7 days), mid term (7–30 days), and long term (> 30 days).

The following adverse events will be included in the review: nerve damage or other neurological injury, vascular puncture, infection at the puncture site, and chronic pain.

### Search methods for the identification of study

We will search the following databases between inception and the date of the search: CENTRAL (The Cochrane Central Register of Controlled Trials), PubMed/MEDLINE (Ovid), CINAHL (Cumulative Index to Nursing and Allied Health Literature), Scopus, and EMBASE. The keywords specific to MEDLINE are available in the Appendix.

### Additional search methods (grey literature)

We will hand-search and screen reference lists of review articles on the topic and of included studies. We will also manually screen conferences proceedings for the last 2 years for the following scientific meetings: Canada Anesthesiologists’ Society Annual Meeting, World Congress on Regional Anesthesia and Pain Medicine, American Society of Anesthesiologists Annual Meeting, Euroanesthesia, and Société Française d’Anesthésie et de Réanimation. Finally, we will search in ClinicalTrials.gov and Who.int for ongoing and unpublished eligible studies.

### Selection of studies

Using the COVIDENCE software (covidence.org), we will remove duplicate records, and two reviewers will independently evaluate titles and abstracts for eligibility. At this stage, we will include any reference deemed eligible by any of the reviewer.

We will then retrieve the full text of potentially relevant studies. Two reviewers will then screen the full text of potentially relevant articles for eligibility. Reasons for exclusion will be recorded. In case of disagreement, the two reviewers will discuss and, if required, a third reviewer will settle the disagreement. If it is necessary to assess eligibility, we will contact the authors to obtain additional information.

### Data extraction and management

Two reviewers will collect data independently and in duplicate on a pretested form including study design, baseline characteristics of the study population, details of the intervention (e.g., type of block, dose of local anesthetic, surgery, utilization of ultrasonography) and comparator, outcomes of interest (definition, unit of measurement and scales), and any miscellaneous data. Divergences will be resolved by consensus or with a third reviewer if needed.

### Assessment of risk of bias

Independently and in duplicate, two investigators will assess the risk of bias of included studies for the primary outcome of this systematic review.

For randomized controlled trials, we will use the Cochrane Collaboration risk of bias tool. The following will be assessed: selection bias, performance bias, detection bias, attrition bias, reporting bias, and any other biases. For selection bias, we will assess the methods used for random sequence generation and allocation sequence concealment. For performance bias, we will assess blinding of study participants and personnel. For detection bias, we will assess blinding of outcome assessors. For attrition bias, we will assess incomplete outcome data generated by withdrawal from a study and loss to follow-up. For reporting bias, we will assess outcomes reported and evaluate for unreported findings by using study registration (ex: ClinicalTrials.gov) or protocol if available. According to specific criteria available in the Cochrane Collaboration risk of bias tool, the two reviewers will categorize the bias as “low risk of bias,” “unclear risk of bias,” and “high risk of bias” [17].

For observational studies, we will use the risk of bias tools developed by the CLARITY (Clinical Advances through Research and Information Translation) group at McMaster University. For cohort studies, the 8 following elements will be assessed: (1) exposed and non-exposed cohorts are drawn from the same population, (2) confidence in the assessment of the exposure, (3) absence of an outcome of interest at the start of the study, (4) exposed and non-exposed cohorts matched for all variables or statistical adjustment, (5) confidence in the assessment of the presence or absence of prognostic factors, (6) confidence in the assessment of the outcome, (7) quality of follow-up, (8) similarity in co-interventions between the groups. For each aspect, the reviewers will categorize the bias as “definitely yes,” “probably yes,” “probably no,” and “definitely no” [18].

For case-control studies, the five following elements will be assessed: (1) confidence in the assessment of the exposure, (2) confidence that the case has developed the outcome of interest and the control has not, (3) the proper selection of cases, (4) the proper selection of the control, and (5) the adequate matching between case and controls according to significant prognostic variables or statistical adjustment. For each aspect, the reviewers will categorize the bias as “definitely yes,” “probably yes,” “probably no,” and “definitely no” [19].

### Dealing with missing data

We will contact the study authors if data relevant to the systematic review are missing in the study report. If they fail to reply within 2 weeks of our first contact and after one reminder, we will acknowledge the missing data and proceed with the analyses.

### Data synthesis

#### Measures of treatment effect

Functional recovery following surgery may be evaluated using different scales [5]. We will use the approach described by Thorlund et al. to enhance the interpretability of the functional recovery outcomes which consists of converting to units of the most familiar instrument the estimates derived from the pooled standardized mean difference or from the individual trial summary statistics, making comparison and analysis more feasible [20].

We will use a random effects model to pool the relevant RCTs if appropriate. The results will be presented as relative risk with 95% confidence intervals (CI) for dichotomous outcomes and as mean difference for continuous outcomes with 95% CI.

##### Assessment of reporting bias

If more than ten studies are included in the meta-analysis, we will evaluate for publication bias by visually inspecting the funnel plots. If publication bias is suspected based on funnel plot inspection, we will detect funnel plot asymmetry with the Egger test for continuous data and the arcsine test for dichotomous data [17, 21, 22].

##### Subgroup analysis and assessment of heterogeneity

We will assess for heterogeneity between studies using the chi-squared test for homogeneity, where *p* < 0.10 indicates substantial heterogeneity, and the *I*^*2*^ statistic. Irrespective of the degree of heterogeneity, we will perform a limited number of subgroup analyses evaluating interaction between study-level subgroup-defining variables and the intervention (i.e., brachial plexus block).

We will perform the following subgroup comparisons, in the meta-analysis, if we find more than four studies on the subgroup comparisons.

1. Type of brachial plexus approach: interscalene vs supraclavicular vs infraclavicular vs axillary, knowing that the success rate is not the same for each approach and hypothesizing that the axillar approach as the highest success rate, thus influencing the success rate of the procedure and the need for concomitant additional anesthetic blockade or GA.
2. Duration of action of the local anesthetic used: short and intermediate acting (chloroprocaine, lidocaine) versus long acting (ropivacaine, tetracaine, bupivacaine), hypothesizing that longer acting anesthetics provide better pain and control and improve functional recovery.
3. Type of surgery: ORIF vs other surgery, hypothesizing that level of function before surgery may not be the same before the procedure, better for a patient presenting with an ORIF for an acute fracture than a patient having a chronic injury, thus having an impact on the level of function after the surgery and the starting point of recovery.
4. Hand, wrist, elbow, or shoulder injury for multiple years with a more significant reduction in range of motion and therefore having an impact on the level of function after the surgery and the starting point of recovery.
5. Year of the study: study conducted before 2008 vs after 2008, hypothesizing the evolution of peripheral anesthesia has improved the success rate of peripheral nerve block as compared to GA.

##### Sensitivity analyses

Sensitivity analyses will be performed excluding studies only reported as abstracts.

#### Narrative synthesis

The narrative synthesis will be organized in the following categories, as recommended by Petticrew and Robert: (1) study issues, (2) study design, and (3) quality of the study and/or the intervention focusing on the effects and the factors impacting the implantation. We will describe the results for each study including the risk of bias assessment [23].

### Assessment of the quality of evidence

To assess the certainty of evidence of each individual outcome, we will use the Grading of Recommendations, Assessment, Development and Evaluation (GRADE) approach. The GRADE approach specifies four levels of quality: high, moderate, low, and very low. RCTs start as high-quality evidence. However, the presence of one or more of the following factors may decrease the quality level of evidence: (1) limitations in the design and implementation of available studies (individual study risk bias), (2) indirectness of evidence (indirect population, intervention, control, outcomes), (3) unexplained heterogeneity or inconsistency of results, (4) imprecision of results, and (5) publication bias. Data from observational studies start as low-quality evidence, but can be upgraded in the presence of the following factors: (1) large magnitude of effect, (2) dose-response gradient, and (3) all plausible confounding would reduce a demonstrated effect or suggest a spurious effect when results show no effect [17, 24].

For each outcome, the findings will be summarized and presented in a table with an explicit judgment of quality of evidence taking into consideration both desirable and undesirable outcomes. An evidence profile will be included in the results showing the GRADE assessments and pooled analysis per outcome.

## Discussion

The improvement of the equipment used for peripheral nerve block has led to an increase of peripheral nerve block in the practice over the last decade. However, evidence supporting the impact of type of anesthesia on patient-important outcomes (functional recovery) is still lacking. Given the theoretical advantages of this technique on early mobilization following surgery, a rigorous evaluation of the available evidence is warranted.

Taking in account the low number of publications on functional recovery of the upper limb, we have decided to include in this review surgery of all the articulations (shoulder, elbow, wrist, and hand). Studies of the impact of peripheral nerve block on the post-operative period are only available for the short-term post-operative period. For the first 7 days, literature shows potential benefits of peripheral nerve block over general anesthesia, but rebound pain can be seen at 24 h, at the end of the effectiveness of the block if the patient does not take any medicine for analgesia. The long-term timepoint is more of an exploratory nature to see if there is an interest in having more information on this timepoint. We are aware that there are many confounding factors in the outcome at the long-term timepoint and will take this in consideration in the analysis of the data.

Strengths of this review include the use of rigorous systematic review methods including predefined study eligibility criteria, a detailed search of both published studies and grey literature, and the possibility of meta-analysis. We expect this study to be limited by the quality of the available studies and the use of heterogeneous tools to evaluate functional recovery.

## Dissemination and knowledge transfer

The systematic review protocol will be registered with PROSPERO, the International prospective register of systematic reviews. We will present the systematic review results at a national conference and publish them in a peer-reviewed journal. The review results will inform the design of a trial comparing RA and GA for upper limb surgery. In addition, the results may increase teaching of peripheral nerve block approach in the anesthesia curriculum and the establishment of workshops to maintain the level of technical skills for practitioners.

## Data Availability

Data sharing is not applicable to this article as no datasets were generated or analyzed during the current study.

## Appendix

**Table 1:** Research strategy

P	“Upper limb surger*” OR “Shoulder surger*” OR “Elbow surger*” OR “Arm surger*” OR “Wrist surger*” OR “Hand surger*” OR “Finger surger*” OR “Radius surger*” OR “Humerus surger*” OR “Acromion surger*” OR “Upper limb procedure*” OR “Shoulder procedure*” OR “Elbow procedure*” OR “Arm procedure*” OR “Hand procedure*” OR “Wrist procedure*” OR “Finger procedure*” OR “Humerus procedure*” OR “Radius procedure*” OR “Acromion procedure*” OR “Upper Extremity Surger*” OR “Orthopedic Procedure*” OR Arthroscop* OR “Upper limb fractur*” OR “Shoulder fractur *” OR “Elbow fractur *” OR “Arm fractur *” OR “Wrist fractur*” OR “Hand fractur*” OR “Finger fractur*” OR “Radius fractur*” OR “Humerus fractur*” OR “Acromion fractur*” OR “Upper limb fixatio*” OR “Shoulder fixatio*” OR “Elbow fixatio *” OR “Arm fixatio *” OR “Wrist fixatio*” OR “Hand fixatio *” OR “Finger fixatio *” OR “Radius fixatio *” OR “Humerus fixatio*” OR “Acromion fixatio*” OR
	MeSH: exp. Upper Extremity/su [Surgery] OR Orthopedic Procedures OR Arthroscopy
I	“Local anaesthesi*” OR “Local anesthesi*” OR “Regional anesthesia” OR “Regional anesthesia” OR “Peripheral anesthesia” OR “Peripheral anesthesia” OR “Nerve block*” OR “brachial plexus” OR “Supraclavicular block*” OR “Axillary block*” OR “Infraclavicular block*” OR “interscalene block*” OR
	MeSH: anesthesia, local OR nerve block OR brachial plexus block
C	“General anesthesia” OR “General anesthesia” OR
	MeSH: Anesthesia, General
O	“Postoperative recovery” OR “Postopertative motion” OR “Postoperative functional” OR “Functional recovery” OR “Quality of Life” OR “treatment outcome*” OR
	MeSH: “Quality of Life” OR treatment outcome

## Abbreviations

CENTRAL: The Cochrane Central Register of Controlled Trials
CI: Confidence intervals
CINAHL: Cumulative Index to Nursing and Allied Health Literature
CLARITY: Clinical Advances through Research and Information Translation
GA: General anesthesia
GRADE: Grading of Recommendations, Assessment, Development and Evaluation
ORIF: Open reduction and internal fixation
RA: Regional anesthesia
RCT: Randomized controlled trials
SFAR: Société Française d’Anesthésie et de Réanimation
VAS: Visual analogue scale
WHO: World Health Organization
